# AI-Driven Drug Discovery: Focus on Targets for Solid Tumors

**DOI:** 10.3390/pharmaceutics18030329

**Published:** 2026-03-06

**Authors:** Jialong Wu, Jide He, Qianyang Ni, Zi’ang Li, Xiushi Lin, Zhenkun Zhao, Lei Qiu, Hongyin Wang, Sijie Li, Chengdong Shi, Yunyi Zhang, Huile Gao, Jian Lu

**Affiliations:** 1 Department of Urology, Peking University Third Hospital, Beijing 100191, China; 2 Key Laboratory of Drug Targeting and Drug Delivery Systems, West China School of Pharmacy, Sichuan University, Chengdu 610041, China; 3 State Key Laboratory of Natural and Biomimetic Drugs, Peking University, Beijing 100191, China

**Keywords:** artificial intelligence, target discovery, solid tumor, machine learning, large language model

## Abstract

In the field of anti-tumor drug development, target identification remains a key component of innovative therapeutic strategies. Solid malignancies have posed significant challenges to conventional target discovery approaches due to their distinct genetic heterogeneity, complex tumor microenvironment, and highly individualized evolutionary trajectories. In recent years, artificial intelligence (AI) has emerged as a revolutionary force in drug discovery. The technological advances from machine learning and deep learning to large language models (LLMs) has enabled the comprehensive integration and analysis of multi-omics biological data and real-world evidence, thereby promoting every stage of the drug discovery process. Thus, this article begins with an overview of the biological characteristics of tumors and the limitations of traditional strategies. It then delves into recent advances particularly in the past three years in the application of AI to drug discovery, especially LLMs. The main focus is on the current landscape of AI-assisted target identification. Furthermore, the article examines key challenges such as multimodal data integration and the interpretability of AI models, and envisions the future path towards integrated AI systems in precision oncology.

## 1. Introduction

Solid tumors represent the major form of malignant neoplasms in adults, accounting for over 90% of all cancer types. They have long been among the most formidable challenges in cancer treatment [1]. Although non-surgical therapies have made continuous progress in recent years [2,3,4], the prognosis of many solid tumors remain suboptimal, especially in advanced stages or those that are highly heterogeneous [5,6,7]. To improve patient outcomes, developing novel anti-tumor agents is a central therapeutic strategy, which, however, is often limited by the lack of effective and highly specific drug targets.

Conventional methods for drug target discovery mostly rely on hypothesis-driven biological research, experimental validation of candidate genes, and retrospective analyses of clinical samples [8]. While these methods have yielded some success, they are less effective when dealing with the complex genetic heterogeneity, and the strong interactions between tumors and the tumor microenvironment (TME) [9,10,11]. Models that focus on linear pathways or single-gene mutations often fail to fully reveal key regulatory nodes, leading to significant limitations in cost, efficiency, and accuracy [12].

With the rapid advancement of high-throughput omics technologies, molecular characterization of tumors has become increasingly refined. Large-scale international databases such as The Cancer Genome Atlas (TCGA), Gene Expression Omnibus (GEO), and the Cancer Dependency Map (DepMap) have collected huge amounts of multi-dimensional biological data, including genomic, transcriptomic, and proteomic layers [13]. Moreover, emerging techniques such as spatial transcriptomics, single-cell sequencing, and whole-slide pathological image analysis also provide researchers with dynamic information about tumors in both spatial and cellular resolutions [14,15]. Due to the data explosion, the research focus has shifted from data acquisition to integration and interpretation, aiming to uncover latent therapeutic and clinical insights. Artificial intelligence (AI), especially machine learning (ML), has become a powerful tool to meet this challenge for its strengths in high-dimensional data processing, feature extraction, and pattern recognition [16]. AI enables the discovery of hidden relationships within complex data, allowing for more efficient and precise identification of drug targets [17]. In recent years, breakthroughs in LLMs have further accelerated AI’s role in drug discovery, significantly improving the workflow from target identification to clinical validation [18].

This review focuses on the critical stage of AI-assisted target discovery in oncology drug development. It begins with a brief overview of the main biological features of solid tumors, followed by a discussion of the multimodal data foundations and common algorithmic frameworks underlying AI-based drug discovery, particularly the practical applications of LLMs. We also examined the representative studies applying AI for target identification in depth. Next, we analyze the current technical challenges confronted by AI approaches, and conclude by exploring the potential of AI in advancing precision cancer treatment, offering insights into future research directions. For literature retrieval, we used keywords such as “large language model”, “LLM”, and “drug discovery” to find recent progress in this area. We also use combinations of “artificial intelligence”, “machine learning”, and “deep learning” with terms like “target discovery”, “target identification”, “cancer”, “tumor”, “neoplasm”, and “oncology” to collect relevant research on AI in solid tumor target discovery.

## 2. Biological Features of Solid Tumors

As a typical form of structured pathological transformation in human tissues, solid tumors evolve through combined effects of multiple factors, rather than a single genetic mutation [9]. This complexity is reflected in their pronounced heterogeneity across molecular, histopathological, and TME levels. These jointly pose a fundamental barrier to the efficacy of targeted therapies. Understanding the core biological traits of solid tumors is therefore essential to define where and how AI can be used in target discovery and offers a theoretical basis for algorithm development.

At the molecular level, the initiation and progression of solid tumors often involve several driver events, including genetic alterations, chromosomal rearrangements and epigenetic abnormalities [19]. A typical example is the formation of the ALK (anaplastic lymphoma kinase) fusion gene, caused by a chromosomal translocation. The aberrant fusion proteins exhibit self-phosphorylation activity, resulting in persistent ALK activation and downstream signaling, thereby promoting cellular proliferation [20]. Researchers have found that circular RNAs derived from EML4-ALK fusion gene variants contribute to tumor cells’ migration and invasion, further complicating the tumor progression process [21]. With the emerging programmed cell death pathways mediated by complex molecular signals, targeting the key regulatory nodes also present promising therapeutic opportunities in tumor suppression [22,23].

From a histological perspective, inter- and intra-tumoral differences in the differentiation status lead to varying prognostic outcomes [9,24]. Taking prostate cancer as an example, some pathological types (such as acinar adenocarcinoma and intraductal carcinoma) can coexist and indicate a poorer outcome [25]. Neuroendocrine prostate cancer, a rare pathological subtype, not only has a poor prognosis but also shows resistance to androgen deprivation therapy due to the absence of androgen receptor (AR) expression, resulting in limited treatment options [26]. Glioblastoma (GBM) is even more complex. While it mainly exists in four major cellular states, approximately 15% of cases simultaneously exhibit two distinct states [27]. Moreover, cells within a single GBM lesion display strong inter-regional gene expression variations [27,28]. These features severely limit the generalizability of traditional drug target design strategies.

**Figure 1:**
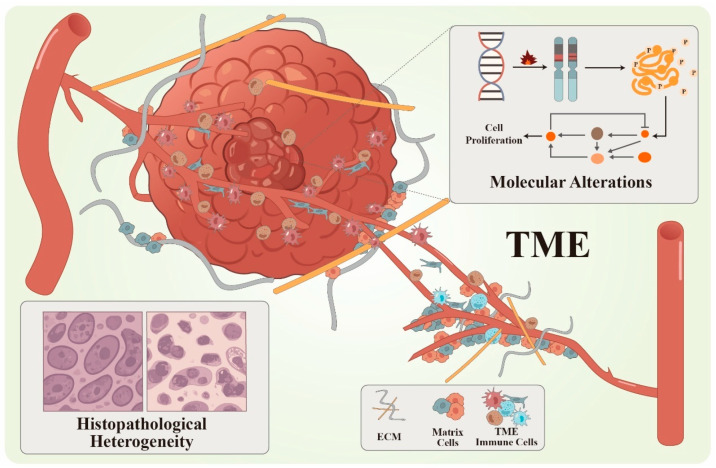
Schematic diagram of the heterogeneous microenvironment of tumors. The figure illustrates the multifactorial complexity of solid tumors, including molecular alterations (e.g., gene mutations and pathway activation), histopathological heterogeneity, and diverse tumor microenvironment (TME) components. These features collectively drive tumor progression and pose major challenges for target discovery and precision therapy. Abbreviations: ECM, extracellular matrix; TME, tumor microenvironment.

The bidirectional interaction between solid tumors and TME is also an important biological clue for target discovery. The TME not only serves as a breeding ground for tumor immune evasion and drug resistance, but also influences cell survival strategies through intercellular signaling [9]. Studies on TME immune components such as tumor-associated macrophages and cancer-associated fibroblasts have illuminated their roles in tumor maintenance [29], which has also broadened the definition of targets. Targets are no longer confined to tumor-intrinsic molecules but now include regulatory factors, signaling mediators, and even spatial structures within the TME (Figure 1). Therefore, in drug development, the definition of a target should extend beyond identifying genetic mutations. It must also consider the functional dependence of tumors on the target and the potential vulnerable links in the entire regulatory network. A good example is synthetic lethality (SL), a genetic phenomenon where the simultaneous inactivation of two genes or pathways leads to cell death [30]. Drugs based on SL principles have been clinically verified across various cancer types, including breast, ovarian, and prostate cancers [31,32,33].

However, against such biological context, the survival outcomes for most patients with advanced solid tumors remain unsatisfactory. This persistent bottleneck reflects the need for the discovery of breakthrough targets that are essential to tumor survival. Inspiringly, AI offers a promising avenue for target discovery, helping to break through the bottleneck in cancer treatment.

## 3. Methodologies of AI in Drug Discovery

### 3.1. Sources of Data

**Figure 2:**
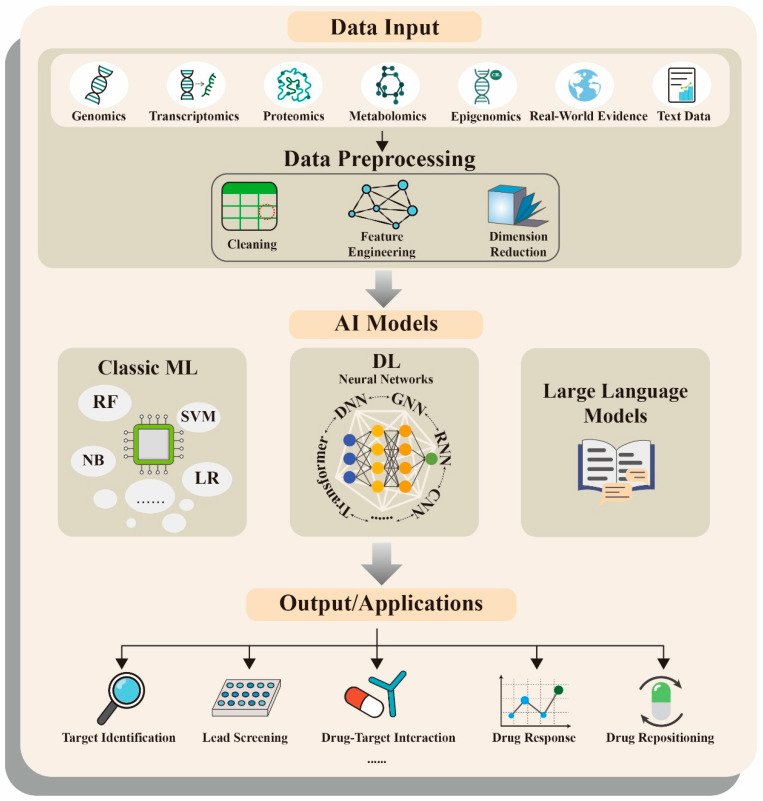
Overview of AI-assisted drug discovery. Diverse biomedical data (multi-omics, real-world evidence, and text) are first preprocessed for model inputs. Classic machine learning (e.g., SVM, RF), deep learning neural networks (e.g., CNN, RNN, GNN, Transformer), and large language models are then used for model construction. The outputs of these AI approaches support key applications such as target identification, lead screening, drug–target interaction prediction, etc. Abbreviations: DL, deep learning; LR, logistic regression; ML, machine learning; NB, naïve Bayes; RF, random forest; SVM, support vector machine.

The first step in AI-assisted target discovery relies on the integration of biomedical data. The rapid growth of omics datasets including genomics, transcriptomics, proteomics, metabolomics, and epigenomics has provided a rich and diverse set of input features for model development [13,34,35,36]. For example, in prostate cancer cohorts like TCGA-PRAD and MSK-IMPACT, researchers can access thousands of samples containing mutation profiles, DNA methylation, and mRNA expressions [37,38,39]. These resources are critical for extracting features of potential therapeutic targets and constructing predictive models. In addition, the vast amount of text from biomedical literature serves as a high-quality corpus for training biomedical-specialized models, which have been successfully applied to target discovery [40,41]. In practical workflows, data preprocessing steps such as normalization, feature extraction, and dimensionality reduction are usually required to improve model trainability and generalization performance. Figure 2 provides an integrated overview of how AI is applied across the drug discovery pipeline, from data input to model output and downstream applications.

### 3.2. Classic Machine Learning Methods

Early ML methods, such as support vector machines (SVM) and random forests (RF), have good interpretability and perform well on datasets with relatively low feature dimensionality, making them suitable for predicting specific targets or pathways [42]. For instance, by utilizing data from TCGA and GEO, Jiang et al. applied least absolute shrinkage and selection operator (LASSO) and SVM-recursive feature elimination algorithms to identify APOC1 as a key gene associated with bone metastasis in prostate cancer, and further validated in vitro [43]. However, with the exponential increase in data dimensionality and volume, traditional ML approaches have shown limitations in handling high-dimensional, large-scale datasets and extracting complex features efficiently [44]. Since feature extraction in these models relies heavily on handcrafted engineering, they often fail to capture nonlinear relationships within the data. Nevertheless, their advantage in interpretability still makes them valuable in certain application scenarios [45,46,47].

### 3.3. Deep Learning

As a major subset of ML, deep learning (DL) has rapidly advanced in the biomedical field due to its ability to automatically learn hierarchical features from complex input data through end-to-end training frameworks. This makes it particularly suitable for analyzing structurally intricate and biologically ambiguous medical data. Deep neural networks (DNNs), the hallmark architecture of DL, have emerged as a dominant paradigm. Convolutional neural networks (CNN), recurrent neural networks (RNN), graph neural networks (GNN), and Transformer, among others, together constitute the core algorithms of deep learning in drug discovery today [48]. For example, the DeepTarget platform employs multi-layer neural networks to integrate protein sequence and structural features, enabling the generation of novel molecules that are structurally distinct from known compounds but share similar activity profiles, based on amino acid sequences [49]. In another study, Snow et al. trained a DNN model using features like AR variant sequences and 2D chemical descriptors, achieving superior predictive accuracy compared to traditional ML methods in determining AR variant responses to AR inhibitors [50]. These examples underscore the growing role of DL in accelerating drug discovery.

CNN and RNN have been widely employed in processing tumor-related sequence data (such as gene and protein sequences) as well as time-series expression data [51,52,53]. In a typical case, Yu et al. developed a hybrid model integrating CNN-RNN, bidirectional long short-term memory (BiLSTM), and DNN to predict potential druggable proteins. Results showed that the model achieved a prediction accuracy of 90%, indicating strong reliability [51]. In another study, Li et al. proposed a generative DL model based on distribution learning, utilizing a conditional RNN framework to construct virtual compound libraries for specific targets [52]. This model was successfully applied to RIPK1, an inflammation-related target involved in necroptosis. Through virtual screening, and in vivo validation, a highly selective inhibitor was discovered, demonstrating the model’s translational potential.

The recent rise of GNN in the field of biomedicine has introduced a new analytical framework for target identification, especially well-suited for the upstream and downstream node-dependent patterns in gene regulatory networks or protein–protein interaction (PPI) networks [54]. By propagating information across nodes, GNN can learn the contextual significance of each node in the entire network, thereby capturing nonlinear dependencies between potential targets and disease phenotypes [55]. For example, Ye et al. integrated biological network data, gene expression profiles, and chemical molecular structures into a heterogeneous graph, which was then processed using a GNN-based model. This target prediction tool, KGDRP, demonstrated a higher success rate in identifying drug targets and candidate compounds compared to previous methods [56]. In addition, some scholars have also optimized the GNN algorithm to enhance its learning ability for 3D structures, significantly improving the performance in predicting drug–target affinities [57,58]. Meanwhile, PocketMiner, an optimized geometric vector perceptron-GNN introduced by Meller et al., enables fast and accurate prediction of cryptic binding pockets, substantially expanding the druggable proteome over simulation-based methods [59]. The advantages of GNN in handling graph-structured data make it an indispensable part in multimodal learning. Xia et al. first utilized a GNN-based model to extract molecular graph features, and subsequently integrated these with SMILES (Simplified Molecular Input Line Entry System) strings, tabular gene expression data, and vectorized molecular fingerprints through self-attention mechanism. This integration formed the basis of the TransCDR model, which accurately predicted the sensitivity of cancer cells to unknown drugs [60]. Similarly, MultiCTox models employed comparable multimodal data and processing strategies to assess drug toxicity and safety profiles, yielding promising performance [61]. Such research highlights the unique advantages of GNN in complex network analysis.

The Transformer architecture, which relies entirely on attention mechanisms, has gained increasing traction in learning tasks involving complex feature interactions due to its ability to dynamically capture long-range dependencies across different modalities. In drug–target interaction (DTI) prediction, Transformer is widely applied to go beyond the limitations of linear SMILES representations and comprehensively capture structural features of drug molecules, identifying potential interactions between distant sites [62]. Monteiro et al. proposed DTITR, an end-to-end Transformer-based model that integrates self-attention encoders and a cross-attention module to simultaneously capture biological, chemical, and pharmacological contexts, thereby significantly enhancing both performance and interpretability [63]. Nevertheless, the accurate representation of drug structures still limits the performance improvement [64]. To address the issue, Zhou et al. used a variational autoencoder (VAE) to encode molecular structures and constructed a Transformer-based model named TransVAE-DTA, which demonstrated improved performance in DTI prediction [65]. In addition, Monteiro et al. further extended the application of Transformer to de novo drug design [66]. By integrating a Transformer-based generator and predictor with feedback-driven multi-objective optimization mechanism, they achieved molecule generation without the need for property-specific labeled datasets. Some researchers have also combined graph Transformer with GNN to preprocess molecular graphs, yielding notable performance improvements [67]. It is worth noting that the Transformer architecture forms the backbone of current LLMs. Its attention mechanism not only revolutionized natural language processing but also laid the methodological foundation for LLMs’ expanding role in drug discovery.

## 4. Large Language Models

Since 2023, LLMs, exemplified by GPT-4, have achieved groundbreaking progress in natural language processing. Their powerful capabilities in language comprehension and generation are gradually being extended to complex and highly structured scientific tasks, including drug discovery [18]. Table 1 summarizes the application progress of LLMs in the field of drug discovery over the past three years. It is important to note that drug development differs fundamentally from pure text-based tasks, as it involves specialized knowledge such as molecular structure interpretation, reaction logic reasoning, and pharmacological modeling. As a result, LLMs require certain adjustments for real-world applications; otherwise, it will face serious “hallucinations”, producing hallucinations and inability to process chemical representations. To address these limitations, researchers have begun integrating LLMs with external tools, a trend facilitated by the availability of application programming interface access in most commercial models. For instance, ChemCrow enhances GPT-4 by linking it with 18 domain-specific chemistry tools through interfaces like LangChain. This integration significantly improves the accuracy, interpretability, and generalization of LLMs in drug discovery tasks [68]. This paradigm has inspired further studies to explore and redefine the boundaries of LLM applications in pharmaceutical research.

Zhou et al. introduced an alternative approach with the development of the TSMMG model, which adopts a “teacher-student” framework [69]. A suite of expert tools and models function as “teachers”, extracting specialized knowledge including molecular structures, physicochemical properties, target affinities, and ADMET (absorption, distribution, metabolism, excretion, toxicity) characteristics. These insights are then converted into natural language descriptions corresponding to the molecular sequences, forming a large-scale text–molecule paired dataset. TSMMG, acting as the “student”, is trained on this dataset, enabling it to handle multiple constrained tasks without the need for repeated fine-tuning. It demonstrated better performance compared to ChemCrow and exhibited strong generalization capabilities, particularly in generating novel molecules with multiple complex properties. Notably, the teacher–student training loop allows for bidirectional improvement, whereby molecular generation by the student model can also refine the teacher modules, forming a self-reinforcing optimization cycle.

In molecular structure design, conventional methods often rely on GNN or diffusion models to process 3D information. However, 3DSMILES-GPT innovatively encoded the 3D structure of molecules as a discrete token sequence. This enabled the use of a token-only large language model to generate SMILES strings along with corresponding atomic coordinates that were capable of reconstructing 3D structures [71]. This method achieved a threefold increase in generation speed while maintaining molecular druggability and synthetic accessibility, effectively balancing between precision and efficiency. Moreover, the model’s interpretability module offers new opportunities to explore the mechanisms of DTI. In comparison, FragGPT focused on the optimization of molecular fragment assembly. It re-encoded complex molecular structures and employed a pretraining plus low-rank adaptation (LoRA) strategy for multitask adaptation [72]. By incorporating reinforcement learning and ADMET-guided constraints, FragGPT achieved significant performance in tasks such as linker design, side-chain modification, and scaffold hopping. FragGPT demonstrated greater flexibility and controllability particularly in multi-objective and multi-constraint drug design scenarios, positioning it as a potential core engine for unified molecular generation platforms.

Apart from structure generation, lead compound optimization represents another key application of LLMs. Ye et al. developed DrugAssist, an interactive molecular optimization platform based on LLaMA2-7B-Chat, which was fine-tuned and LoRA-adapted using a custom-built large-scale molecular instruction dataset named MolOpt-Instructions [77]. The model enables users to specify optimization goals via natural dialogue. It also supports iterative refinement based on human feedback following unsuccessful attempts. DrugAssist outperforms BioMedGPT and native ChatGPT (GPT-3.5-turbo) in terms of success rate, controllability, and generalization across multi-property optimization tasks. It is particularly effective in scenarios requiring to meet specific numerical constraints, providing a powerful tool for emulating expert-level molecular optimization.

Unlike molecule-centric strategies, GexMolGen introduced a phenotype-driven generative framework that bypassed the traditional “target identification-compound screening” process by using gene expression data to guide molecular generation [74]. The model employed scGPT, a single-cell LLM, as the gene encoder, and integrated with a graph-based hierVAE model as the molecular decoder. Through a cross-modal “first-align-then-generate” strategy, expression features are embedded into a unified space, enabling molecule generation for specific expression patterns. The framework demonstrated strong zero-shot generalization and biological consistency in gene-knockout and cross-transcriptomic tests, making it particularly suitable for complex, heterogeneous diseases like solid tumors.

Importantly, the utility of LLMs extends beyond structure design to higher-level tasks such as drug repurposing. DrugReAlign enhanced GPT-4’s performance by embedding expert knowledge such as target annotations and mechanisms of action into natural language prompts, thereby improving prediction accuracy and interpretability in drug repositioning tasks [75]. This successfully mitigates the “hallucination” problem. Similar strategies have been adopted in general LLMs like Claude 3 Opus, where researchers used plain-language prompts (e.g., “incorporating electron donating group by tweaking only the side chains”) to guide molecular modifications, achieving up to 97% effectiveness and novelty without any fine-tuning, greatly lowering the threshold of molecular design [76].

In early-stage drug discovery for osteosarcoma, LLMs have also been applied to real-world screening tasks. One study utilized GPT-4 to predict the anticancer activity of 60 natural polyphenols by modeling the relationship between structural features (e.g., number of aromatic rings, hydroxyl groups, and hydrophobicity) and in vitro IC_50_ values. Gossypol was successfully identified as a promising lead compound against osteosarcoma and further assembled into a nanoparticle delivery system that showed strong antitumor effects in an orthotopic model [70]. Although this study relied on manually input structural parameters rather than automatic analysis, it still highlights the unique role of LLMs in rational structure–activity relationship analysis.

## 5. AI-Assisted Target Discovery in Solid Tumors

In the advancement of precision medicine for solid tumors, efficient identification of therapeutic targets has become pivotal for achieving personalized treatment. In tumor types where conventional methods have significant limitations, AI have demonstrated clear advantages, enabling the identification of several novel, biologically relevant targets that accelerate anti-cancer drug development (Table 2).

With the development of single-cell multi-omics technologies, cell surface proteins are gaining increasing attention for their roles in cell identity characterization and drug target development. However, traditional experimental approaches are constrained by antibody availability and cost, resulting in the detection of only a small subset of the theoretical cell surface proteome [85]. To address this limitation, Chen et al. proposed the SPIDER model, a deep ensemble learning and zero-shot learning framework with context-agnostic design [80]. By integrating six independent CITE-seq datasets across diverse tissue types and disease states, SPIDER accurately predicts the abundance of over 2500 surface proteins, exhibiting strong cross-tissue and cross-disease generalizability. While the model exhibits high reproducibility through its open-source code and standardized benchmarks, its reliance on existing CITE-seq antibody panels for training introduces an inherent technical bias. The “zero-shot” prediction of unseen proteins assumes that RNA-protein correlations are consistent across the surfaceome, potentially overlooking targets governed by complex post-transcriptional regulation. In addition, despite the computational strengths, the study remains primarily an in silico tool, lacking the direct wet-lab validation, which is necessary to confirm the therapeutic relevance of its predicted markers.

Other studies have emphasized the discovery of TME-related targets that are associated with therapeutic response. In intrahepatic cholangiocarcinoma, Ji et al. developed a radiotranscriptomic prediction model that integrated spatial transcriptomics with contrast-enhanced CT imaging via ML [78]. This model generated an immune risk scoring system and identified PLAUR (uPAR) as a key target in high-risk patients. Researchers utilized patient-derived tumor xenograft models to demonstrate that anti-uPAR antibodies synergize with anti-PD-1 therapy, showcasing significant translational potential by moving beyond correlation to functional evidence. However, the cohort used for predicting immunotherapy response (*n* = 36) was notably small, which may lead to an optimistic estimation of the reported AUC (0.84) and raises concerns regarding overfitting. Such sample size limitations are a recurring challenge in radiogenomics, where high-dimensional feature spaces often collide with sparse clinical cohorts. Additionally, Ager et al. proposed an immune feature recognition strategy that combined high-parameter flow cytometry with ML analysis [84]. Using time-series sampling from murine prostate cancer (NPK-C1) and colorectal cancer (MC38) models, they trained a RF classifier to construct a KLRG1 regulatory module. This enabled the identification of KLRG1^+^ CD4^+^ T cells as a crucial subgroup associated with tumor burden and immune evasion. In single-cell data of human clear cell renal cell carcinoma, the KLRG1 module score was significantly higher in tumor tissues than in normal and increased with disease progression. Nevertheless, its translational potential as a therapeutic target remains speculative, as the work lacked functional blockade or knockdown experiments to prove that neutralizing KLRG1 can indeed rescue immune exhaustion. AI has also expanded the “antigen discovery” toolkit. Liao et al. developed a multi-factorial integrated model (MARS) based on mass spectrometry data that can successfully identify non-canonical MHC-I-presenting peptides, including neoantigens derived from lncRNAs, without relying on RNA or DNA sequence information [82]. This approach overcomes the shortcomings of traditional de novo antigen prediction in specificity and accuracy, particularly improving recognition for challenging alleles such as HLA-B27. Collectively, these works provide an efficient ML-driven solution for target discovery in tumor immunotherapy.

In addition to structured biological data, some researchers have also attempted to utilize unstructured textual evidence from literature. Liu et al. developed a probabilistic knowledge graph framework named Progeni, which integrates heterogeneous biological networks with literature-derived evidence, and employs GNN to model and predict relationships among biological entities [41]. Through literature mining and in vitro validation, Progeni successfully identified and confirmed several novel targets related to melanoma and colorectal cancer. However, it needs to be completely retrained each time it uses a new dataset, which imposes a substantial computational burden and limited scalability [41]. Integrated AI platforms have served as viable solutions to this challenge. PandaOmics is a representative example, incorporating 23 distinct scoring strategies, including omics-based features, network proximity, and text mining, to systematically analyze multi-omics data from over 16,000 healthy tissues and 11,000 solid tumor samples [83]. The platform identified 22 candidate genes, such as KDM1A and PARP1, with dual anti-cancer and anti-aging potential, and validated their function in animal models. Similarly, the BenevolentAI platform utilized a large-scale knowledge graph built from over 35 million scientific publications and numerous databases. It applies tensor factorization machine learning and causal inference algorithms to prioritize candidate targets that can selectively kill platinum-resistant ovarian cancer. This approach identified the TNIK-CDK9 axis as a core survival mechanism in platinum resistance, and validated the compound NCB-0846 as an effective inhibitor [79]. These examples underscore the feasibility of leveraging diverse AI-based strategies for target discovery. It should be noted that although PandaOmics and BenevolentAI utilize natural language processing techniques in knowledge extraction, their core architectures are not based on LLM. Instead, they rely on structured knowledge graphs and causal reasoning frameworks, which fundamentally differ in task objectives and algorithmic design from the current language generation-based LLMs.

Despite the powerful performance and high biological validation of PandaOmics and BenevolentAI platform, the commercial and “black-box” nature of these platforms hinders independent verification. Without access to the underlying model weights or training scripts, the scientific community cannot easily discern whether these predictions are truly novel or merely reflections of “literature bias”, where algorithms favor well-studied pathways over obscure but potentially transformative targets. In contrast, the Progeni framework [41] and the MARS method for immunopeptidomics [82] offer higher transparency by providing open-access code or detailed mathematical formulations. Specifically, MARS addresses a critical gap in neoantigen discovery by successfully predicting non-canonical MHC-I peptides that elicit CD8^+^ T-cell responses in healthy donors. This transition from computational prediction to functional T-cell activation represents a milestone for translational AI, even if the model’s performance fluctuates across different HLA alleles, which reminds us of the persistent challenge of dataset imbalance in specialized omics.

## 6. Discussion and Future Outlook

AI is permeating every aspect of life and work, and drug discovery is no exception. The integration of AI tools has brought transformative methodological advances across the entire drug development pipeline, including target identification, lead compound screening [86,87], DTI prediction [88,89], and drug repurposing [90], offering powerful means to address the challenges posed by tumor heterogeneity in anti-tumor drug development [91]. By reviewing existing literature, it can be seen that massive multi-omics data and biomedical literature materials form the data basis for AI modeling. This article first outlines classical ML approaches and then highlights advances in neural network-based DL. Special emphasis is placed on the application of LLMs, which represent a paradigm shift in the field. We then systematically summarize recent progress in AI-assisted target discovery for anti-tumor drug development. Collectively, these findings demonstrate that AI is profoundly reshaping the landscape of drug discovery, driving the construction of a new framework for drug research and development.

Nowadays, cancer treatment has embraced the concept of precision medicine [92], where the development of individualized therapies depends on the identification of effective therapeutic targets. AI has emerged as a trustworthy “wingman” in this process. Early efforts include the BANDIT model developed by Madhukar et al., which centers on Bayesian inference and integrates chemical structures, pharmacologic profiles, and gene expression data to identify DTI [47]. Notably, BANDIT successfully predicted DRD2 as the target of ONC201, resolving the issue of identifying targets of mechanism-unknown small-molecule agents. Lin et al. combined supervised and unsupervised learning to analyze cfDNA and identified 16 prostate cancer targets linked to treatment resistance [46]. The CLIM platform, leveraging diverse ML techniques, discovered that loss of UQCR11 induces dependency on MTHFD2 in ovarian cancer, revealing a collateral lethality mechanism, which broadened the target recognition space beyond SL [45]. It can be seen from recent studies that conventional ML continues to play a key role in target discovery with remarkable success. This may be because the performance of ML methods is temporarily sufficient to meet the current task requirements and has strong interpretability, allowing researchers to understand the importance of top-ranked features and validate them through biological methods. However, the limitations of ML should also be clearly identified.

1. Insufficient utilization of multi-omics data. Most ML applications still rely heavily on genomic and transcriptomic sequence data. Even in Ji et al.’s innovative model that fuses enhanced CT imaging and spatial transcriptomics, only image-derived features were used in training with spatial transcriptomics serving mainly as labels to improve interpretability [78]. Additionally, omics feature extraction for ML often requires hand-crafted features, requiring highly specialized expertise and a large amount of work.

2. Inadequate handling of multimodal data. In drug discovery, essential data sources also include molecular graphs, biomolecular interaction networks, and knowledge graphs. Yet, traditional ML is ill-equipped for graph-structured data or unstructured text from scientific literature. GNN, such as in the Progeni model [41], have shown promise in addressing this gap. Alternatively, integrated AI platforms like Benevolent and PandaOmics have been developed to handle multimodal data in target discovery [79,83]. Additionally, the advantage of DL in integrating multi-omics and multimodal data for predicting drug targets is also well demonstrated in Chen’s research [80], and is applicable to other stages of drug discovery [60,61,93].

Of note, few models to date can directly train on digital pathological whole-slide images due to their massive resolution and complexity. However, the Transformer-based GigaPath model recently overcame this challenge by introducing a sparse attention mechanism (LongNet), greatly enhancing the global context modeling in histopathological images [94]. The pre-trained Prov-GigaPath on real-world large-scale datasets is expected to bring significant breakthroughs in the integrated analysis of pathological images and other modal data.

One of the major limitations of DL lies in its “black-box” nature, which severely constrains interpretability, especially in deeply nested architectures. Therefore, many researchers have attempted to improve interpretability through algorithm optimization or model design. In a 2021 study by Elmarakeby et al., the P-NET model incorporated biologically informed priors, such as genes, pathways, and biological processes, into a hierarchical neural network structure [95]. All connections were derived from known biological relationships, endowing the model with intrinsic interpretability. Attribution methods like DeepLIFT and visualization techniques were used to trace prediction paths back to specific molecular features and pathways. It successfully identified novel targets in prostate cancer [95]. This method of embedding prior biological knowledge to build self-explainable models has later been recognized as an important means to enhance model transparency [96,97].

However, such architecture-driven interpretability depends heavily on the quality and completeness of pathway databases (e.g., Reactome used in P-NET). If prior knowledge is inaccurate or incomplete, model outputs may be misled or miss critical information. Additionally, rigid reliance on predefined pathways may render the model unable to explore novel mechanisms freely. In contrast, the Progeni model improves internal interpretability by introducing literature-supported probabilistic edge weights, providing a balance between biological reasoning and model flexibility [41]. It is important to clarify, however, that empirical validation of model predictions does not equate to logical transparency. A model might mistakenly take an irrelevant feature, originated from spurious correlations in the data, as a prediction basis, and still yield consistent predictions by coincidence. While downstream wet-lab experiments can be used to examine results, which predispose us to lay more emphasis on its actual effects (e.g., an inhibitor targeting a certain prediction target can indeed inhibit tumor growth), without internal model interpretability, the discovery process risks reverting to the inefficient “hypothesis-experiment” path. Therefore, truly interpretable models are essential for improving efficiency and reducing cost. Such models should simultaneously satisfy both internal interpretability (clarity of decision logic) and external verifiability (alignment with experimental or clinical results), ensuring robustness against data coincidence and bias.

Regrettably, the application of LLMs in target discovery remains significantly underdeveloped, with current efforts primarily focused on lead compound screening and optimization. This disparity likely stems from the inherent complexity of target discovery, which demands higher requirements for causality reasoning, cross-modal integration, and mechanistic modeling, while current LLMs are mainly optimized for associative reasoning and sequential language generation. Therefore, LLMs are not yet fully equipped in this field. In contrast, compound screening tasks typically involve well-defined structural inputs (e.g., SMILES) and continuous supervision signals, making them more amenable to LLM-based optimization. Based on this, recent research has begun exploring ways to enhance LLM performance and adaptability for complex drug discovery tasks. Among the critical determinants of an LLM’s performance is its parameter count, typically fixed by the pre-trained model itself. For example, LlaMA2-7B-Chat contains approximately 7 billion parameters, whereas mainstream models like GPT-4 are estimated to have several orders of magnitude more. 

Despite the superior language comprehension and generation of GPT-4 and similar models, their application in domain-specific scientific tasks remains limited due to differences in training corpora and modeling approaches. To address these flaws, researchers have increasingly adopted strategies such as modular augmentation and lightweight adaptation. For instance, ChemCrow integrates domain-specific databases, analytical tools, and knowledge graphs to provide GPT-4 with appropriate context and causal reasoning support [68]. LoRA has emerged as a popular fine-tuning method that allows the introduction of only a small number of parameters for task-specific training without altering the base model, and has been successfully applied in models like DrugAssist and FragGPT [72,77]. Meanwhile, prompt engineering has also become a central means for aligning model outputs with task requirements. In the LLMPN model, structured natural language prompts incorporating pharmacological mechanisms, target classes, and modes of action significantly improved the biological plausibility of predicted targets [70]. Similar strategies have been adopted in TSMMG and Claude 3 Opus as well [69,76]. These cases indicate that prompt engineering is not only crucial for guiding LLMs to “understand task intent”, but also serves as a low-cost, generalizable customization approach, especially when direct model fine-tuning is impractical. Interestingly, researchers have even exploited LLMs’ probabilistic “hallucination” tendencies to generate novel hypotheses. By prompting GPT-4 to hypothesize drug combinations that are selectively cytotoxic to breast cancer cells, several effective drug pairs were identified [98]. This suggests that LLMs can not only promote the efficiency of scientific research, but also function as innovative and intelligent tools in drug discovery. Overall, the advantages currently demonstrated by LLMs in drug discovery mainly lie in the following aspects: 1. Through prompts, dialogue-based inputs, or conditional instructions, LLMs can be adapted to diverse objectives ranging from simple property control to complex molecular design; 2. LLMs outperform most traditional approaches in novelty and generalization capabilities when generating molecules or hypotheses; 3. Their end-to-end modeling capabilities from molecular to natural language representations tremendously enhances task flexibility and controllability when combined with simple human–computer interfaces. 

However, it is critical to recognize that AI-driven drug discovery still faces several challenges before fully translating to clinical practice. Despite the explosive growth of omics data, most studies do not fully utilize multi-omics and multimodal data, partly due to difficulties in data accessibility and cross-platform standardization. Collective analysis of aforementioned studies reveals an inevitable concern that the performance frequently drops when models are moved from internal cross-validation to independent external cohorts. This often stems from insufficient cross-dataset consistency. Significant heterogeneity in sample preparation, sequencing platforms, and batch effects between public databases (e.g., TCGA) and proprietary institutional datasets can introduce non-biological noise, causing models to learn dataset-specific artifacts rather than universal tumor biology [99,100]. To address data heterogeneity and distributional misalignments in preclinical drug modeling, Parrondo-Pizarro et al. developed AssayInspector, a systematic tool for data consistency assessment (DCA) [101]. By utilizing this tool to analyze public datasets, the authors revealed significant distributional differences and annotation inconsistencies between popular benchmarks and gold-standard data sources, noting that these discrepancies primarily stem from variations in experimental conditions and chemical space coverage [101]. Informed data integration strategies based on DCA help identify outliers and batch effects more effectively, thereby avoiding the noise introduced by direct data standardization or integration. This ultimately enhances the predictive reliability and generalizability of machine learning models [101], which offers a promising direction for mitigating performance drops.

Nevertheless, harmonizing data is merely a technical prerequisite. The conceptual leap from statistical association to causal inference remains a more formidable challenge in target discovery. In a systematic review of over 100 studies on immunotherapy research, Wang et al. found that none employed causal inference methods [102]. The neglect of causal inference resulted in severe and misleading consequences. For instance, without correcting for immortal time bias, a traditional model falsely linked immune-related adverse events with better survival [102]. Such spurious correlations, driven by unmeasured confounders and methodological flaws, not only undermine the biological plausibility of these models but also contribute to the exclusion of numerous predictive algorithms in phase III clinical trials [102]. In the complex, non-linear signaling networks of solid tumors, a gene highly correlated with disease progression may merely be a downstream effector or a “passenger” event rather than a driver essential for tumor survival. Shi et al. revealed through a comprehensive evaluation that although certain algorithms perform relatively well, single computational methods struggle to distinguish cancer driver genes from passenger genes without strict threshold constraints [103]. Consequently, targets predicted based solely on association often lack the functional necessity required for therapeutic intervention, leading to high failure rates in downstream experiments. The collective evidence suggests that without integrating causal inference into AI architectures, models will fail to identify true biological drivers, ultimately hindering clinical translatability. This challenge is highlighted by Qu et al. in their work on pancreatic cancer diagnosis, where the integration of causal intervention successfully eliminated spurious correlations [104].

Furthermore, the transition from in silico predictions to clinical reality is hindered by reproducibility issues and regulatory expectations. The “black-box” nature of deep learning, combined with algorithmic stochasticity, can lead to inconsistent target prioritization across model runs [105,106], undermining confidence for wet-lab validation. Cases where models learn spurious correlations exemplify this fragility, as described by Ball, where models diagnosed diseases based on image artifacts rather than genuine pathology [107]. This lack of robustness directly conflicts with evolving regulatory standards: the FDA has released Artificial Intelligence/Machine Learning Action Plan since 2021, requiring transparency and clinical validation [108]. Without a clear audit trail distinguishing true targets from spurious correlations, opacity creates significant barriers to clinical adoption. This concern is underscored by Liu et al., who note that the algorithmic “black box” nature may make it difficult for clinicians to understand the predictive basis [109]. Further interpretability challenges arise from high-dimensional data complexity, inherent network biases toward well-studied proteins, and a reliance on “average patient” models that overlook disease heterogeneity and subtypes [109]. While simple linear models like Elastic Net-regularized CoxPH [81] offer high interpretability and have successfully identified survival-associated ion channels in glioblastoma, the field is increasingly gravitating toward complex neural networks and ensemble architectures. This shift necessitates a careful trade-off: as models become more capable of capturing non-linear biological complexities, they often compromise the interpretability required for clinical trust. Bridging this gap to achieve the dual goals of internal interpretability and external verifiability requires a standardized framework encompassing open-source code for reproducibility, multicenter external validation to mitigate bias, and functional wet-lab verification to ensure clinical translatability. The value of such external validation is underscored by Christiansen et al., who validated their model across 8 countries, 20 centers, and 21 ultrasound systems [110]. Finally, this review primarily focuses on AI-assisted target discovery in oncology, while methods developed for non-cancer indications may also be applicable in cancer drug discovery, which poses a limitation to the generalizability of the current summary.

In summary, with the continuous progress of large models, computing power, and algorithm design, AI will keep driving the innovation of drug development, especially in the field of precision oncology. From early predictive tools to becoming a central driver of drug discovery, AI-driven target discovery will play an increasingly vital role in expediting therapeutic development, improving efficiency, and reducing the burden of disease for patients.

## Figures and Tables

**Table 1:** LLMs: Emerging Applications in Drug Discovery.

Stage	Model	Architecture	Modalities	Tasks	Performance	Year	Ref.
Lead discovery	TSMMG	GPT-2 (pre-trained and prompt optimized)	Text (text–molecule pairs)	Acted as a “student” LLM, learning to generate novel molecules from natural language descriptions by distilling knowledge from “teacher” models, satisfying multiple constraints via text prompts	Parameter size: GPT-2 level	2025	[69]
Lead screening	LLMPN	GPT-4 (task-specific prompt optimization)	Text (chemical descriptors), numerical (IC50), structural (IUPAC)	Used to analyze polyphenol structure–activity relationships, adapted through task-specific prompts and structured molecular descriptor inputs, to identify effective lead compounds for osteosarcoma	Parameter size: GPT-4 level; gossypol predicted as top candidate from 60 polyphenols	2025	[70]
Lead optimization	ChemCrow	GPT-4 (ReAct/MRKL agent framework with tool augmentation via LangChain)	Text, structured chemical representations (SMILES, CAS)	Adapted GPT-4 into a chemistry-aware agent by integrating 18 chemistry tools to autonomously perform synthesis planning and compound screening	Parameter size: GPT-4 level; successful automated synthesis of 4 compounds	2024	[68]
Lead screening & optimization	3DSMILES-GPT	Transformer decoder (8 layers, 12 heads)	2D SMILES, 3D atomic coordinates, protein pocket surface	Adapting a token-only LLM through pretraining, protein-aware fine-tuning, and reinforcement learning to generate high-affinity, drug-like, and synthesizable 3D molecules	Parameter size: not provided; achieved strong prediction ability and 3 times faster generation speed	2024	[71]
Lead optimization	FragGPT/FragGPT-ADMET	GPT-2 (fine-tuning with LoRA)	Text-based molecular fragments (FU-SMILES)	Fragment-based molecular generation optimized via LoRA and reinforcement learning, enabling high-quality and controllable molecular generation across multiple drug design tasks	Parameter size: GPT-2 level; pre-trained on 78M molecules	2024	[72]
Lead screening	GexMolGen	scGPT (integrated first-align-then-generate strategy)	Gene expression (transcriptome), molecular structure (graph)	To generate hit-like molecules from gene expression signatures via a cross-modal framework combining scGPT-based gene encoding, hierVAE-based molecular decoding, and contrastive alignment for modality bridging	Parameter size: scGPT level [73]; achieved 100% validity in molecule generation	2024	[74]
Drug repurposing	DrugReAlign	GTP-4, GPT-3.5, New Bing, medllama3-v20	Text (natural language, structures, spatial interaction, etc.)	Analyzing target sites, generating drug repositioning suggestions, and providing explanations; adapted by multi-source prompts; identified two unrecognized drug–target interactions for cancer therapy	Parameter size: GPT-4 level (best performance of all)	2024	[75]
Lead screening & optimization	Claude 3 Opus LLM	Claude 3 Opus (task-specific prompt optimization)	Text (natural language prompts) and molecular representations (SMILES)	Acted as a molecular design engine for reading, writing, modifying and generating valid and unique molecules; adapted by prompt engineering	Parameter size: Claude 3 Opus level	2024	[76]
Lead optimization	DrugAssist	LlaMA2-7B-Chat (fine-tuned with LoRA)	Text (SMILES strings, natural language instructions)	Fine-tuned with a custom instruction dataset and LoRA to perform molecule optimization, achieving multi-property control, transferability, and expert-guided refinement	Parameter size: 7B; achieved 0.62 multi-property optimization success rate (vs. 0.59 for Transformer)	2023	[77]

IUPAC, international union of pure and applied chemistry; LoRA, low-rank adaptation; SMILES, simplified molecular-input line-entry system.

**Table 2:** Applications of AI on target discovery.

Tumor Type	AI Methodology	Sample Size	Data Type	Data Source	Validation Method	Predicted Target	Performance	Interpretability	Year	Ref.
ICC	ML (logistic regression with RFE)	**Discovery/Training Sets:** 401 pts (Bulk RNA-seq). **Single-cell Data:** 51,642 cells (from 16 ICC pts and 6 normal controls). **Spatial Transcriptomics:** 120 samples from 40 pts (3 anatomical regions each). **Validation Cohorts (*n* = 331):** Molecular (52 pts, 156 specimens), resection (243 pts), and immunochemotherapy cohort (36 pts).	Contrast-enhanced CT (Radiomics), Bulk/Single-cell/Spatial RNA-seq (Multi-omics).	Public (TCGA, GEO, CPTAC) + Institutional Dataset	Internal CV (5-fold) + Independent External Set + In vitro & In vivo validation	uPAR (identified through IRS level)	**Internal:** AUC 0.95 (IRS prediction). **External:** Prognosis: C-index = 0.67 (OS) & 0.64 (recurrence-free survival). Immunotherapy response: AUC = 0.84	High (used interpretable algorithms; selected features show spatial correlation with immune gene expression, confirming biological relevance)	2025	[78]
Ovarian cancer	Integrated AI platform (Benevolent) combining relational inference and causal reasoning algorithm	**N_train:** >35 million scientific articles & databases (Knowledge Graph) + GSE71340 cohort. **N_val:** 13 patient-derived organoids, multiple patient-derived cell lines, and TCGA cohort (*n* = 201 to 307 patients).	Text, structured data (kinase activity profiles, drug compounds, etc.), genomics, clinical and phenotypic data.	Benevolent Knowledge Graph (ChEMBL, Reaxys), GEO, TCGA	Independent External Set + In vitro validation	TNIK, CDK9	Model metrics not reported; prioritized 74 targets from 500 candidates, with 6 hit compounds identified, showing ≥50% cell viability reduction ex vivo.	High (leveraged knowledge graph for transparent relational inference; biological validation via co-expression & pathway analysis)	2025	[79]
PCa	ML (LASSO and SVM-RFE)	**N_train:** 42 samples (GSE77930: 22 PCa, 20 PCa with bone metastasis). **N_val:** 51 samples (GSE32269: 22 PCa, 29 PCa with bone metastasis). **Single-cell data:** 16 pts (9 bone metastasis, 7 normal). **Institutional clinical validation:** 16 pts.	Bulk RNA-seq, scRNA-seq, clinical dataset, and in vitro experimental data.	Public (TCGA, GEO) + Institutional Dataset	Independent External Set (GSE32269) + Wet lab validation (Immunohistochemistry, RT-PCR, Transwell, etc.)	Bone metastasis-related markers (APOC1, etc.)	**Internal:** AUC = 0.727–0.926. **External:** AUC maintained >0.7.	High (relatively interpretable ML models; validated via pathology, functional assays, and GSEA)	2024	[43]
CRC, melanoma	GNN on a prob-KG	**Graph Data (Nodes/Edges):** Dataset 1 (Baseline): 12,015 entities and 1,596,745 associations. Dataset 2 (Wet lab): 27,467 entities and 77,429 associations. **Patient Data (TCGA):** Melanoma: *n* = 176 (metastatic), *n* = 173 (metastatic), *n* = 50 (primary). Colorectal Cancer: *n* = 264.	Heterogeneous biological networks (interactions between proteins, drugs, etc.), unstructured text data, and sequence/structural similarity matrices.	Public (HRPD, DrugBank, PubMed, TCGA, etc.)	Internal CV (5-fold) + Independent External Set (TCGA) + In vitro validation	Novel protein targets in melanoma and CRC	**Internal:** entry-wise AUROC ≈ 0.98 and AUPR ≈ 0.95; cluster-wise AUROC ≈ 0.81 and AUPR ≈ 0.51 **External:** Significant tumor proliferation inhibition; correlated with TCGA patient survival outcomes.	Moderate to High (while GNN-based embeddings remain complex, predicted targets were validated by wet lab experiments)	2024	[41]
HCC, CRC	DNN + ensemble learning	**N_train:** 120,461 cells (6 datasets covering 289 proteins, 5 tissues, 4 diseases, 17 cell types) via 10-fold CV. **N_val:** 4 CITE-seq datasets. **Application Data:** HCC cohort and CRC liver metastasis cohort (125,150 cells).	Single-cell multimodal data (scRNA-seq, cell-type/tissue/disease metadata, surface protein abundance).	Public CITE-seq (GEO and Figshare)	Internal CV (10-fold) + Independent External Set validation (4 distinct cross-context CITE-seq datasets).	Abundance of >2500 cell surface proteins at single-cell resolution	**Internal:** Pearson correlation 0.80 for seen proteins. **External:** Median correlation 0.81 for unseen proteins; superior to baseline models.	Moderate to High (while the ensemble DNN architecture reduced interpretability, the predicted protein abundances were biologically coherent)	2024	[80]
GBM	ML (Elastic net-regularized CoxPH)	**N_train:** 9352 cancer samples across 33 cancer types (150 GBM samples via 1000 iterations of 80% splits). **N_val (External/Lab):** 136 GLASS samples, 55 GEO samples. 3 patient-derived cell lines & mouse cohorts.	Transcriptomic data (bulk RNA-seq), clinical metadata (overall survival, tumor grade/stage, etc.).	Public (TCGA, GLASS, GTEx, GEO, etc.)	Internal CV (Bootstrapping) + Independent External Sets + In vitro & In vivo validation.	GJB2 and SCN9A	Model metrics not reported. **Internal:** High selection frequency with significant OS association. **External:** HR > 1.5 in independent cohorts. Target knockdown significantly prolonged xenograft mouse survival.	High (linear model with interpretable coefficients and Elastic Net enabled sparse feature selection)	2024	[81]
Pan-cancer	Supervised affine-weighted model	**N_Train:** 118,177 spectra (5-fold CV). **N_Val:** ~169 k synthetic peptides; 2424 cancer cell line spectra; 10 primary cervical tumor samples.	Immunopeptidomics (mass spectrometry [MS/MS] spectra of HLA-bound peptides)	Public (PRIDE) + In-house primary tissue data	Internal CV (5-fold) + Independent External Sets (Synthetic & Public) + In vitro validation	Non-canonical MHC-I-associated peptide sequences on tumor cells	**Internal:** FSR improved to 0.782 vs. 0.731 (Baseline). **External:** 90.7% FSR on benchmark; 1.6-fold improvement in recall; FDR < 14.3% at high confidence score.	High (used transparent model with biologically meaningful features; predictions aligned with known immunopeptidomics patterns)	2024	[82]
Pan-cancer	Integrated AI platform (PandaOmics)	**Analysis/Input Set:** 139 cancer datasets (11,303 cases and 4431 controls) + GTEx healthy dataset (16,740 samples from 980 individuals). **Validation Set (In vivo):** 540 C. elegans worms (270 in the RNAi treatment group, 270 in the control group).	Transcriptomics, proteomics, pathway activity scores, literature-derived text, expert and funding metrics.	Public (TCGA, GEO, COSMIC, GTEx, etc.).	In vivo validation (Lifespan experiments in C. elegans via RNAi knockdown).	22 validated dual-purpose therapeutic targets for aging and cancer	Model metrics not reported/applicable; identified 22 dual-purpose targets (e.g., KDM1A) across cancers validated by in vivo experiment.	High (PandaOmics scores integrated biological evidence; predictions were biologically validated)	2023	[83]
ccRCC	unsupervised ML (RF regression, UMAP)	**N_train (Discovery):** Preclinical in vivo models (*n* = 10–25 mice/group). **N_val (Validation):** 2 human ccRCC scRNA-seq cohorts.	34-parameter spectral flow cytometry (protein) & scRNA-seq	Public (GEO) + literature data (previous study)	Independent External Sets + In vivo validation	KLRG1 protein activity in CD4^+^ T cells	Model metrics not reported/applicable; predicted KLRG1 signature showed strong correlation with tumor stage (*p* = 0.0282 for localized vs. normal; *p* = 1.124 × 10^−158^ for metastatic vs. normal)	High (RF provided feature importance scores; predicted KLRG1 activity aligned with tumor progression and known immune phenotypes)	2023	[84]

Abbreviations: AUC, area under the curve; AUROC, area under the receiver operating characteristic curve; AUPR, area under the precision-recall curve; ccRCC, clear cell renal cell carcinoma; CoxPH, cox proportional hazards regression; CRC, colorectal cancer; CV, cross-validation; FDR, false discovery rate; FSR, full sequence recall; GBM, glioblastoma; GNN, graph neural network; GSEA, gene set enrichment analysis; HCC, hepatocellular carcinoma; HLA, human leukocyte antigen; HR, hazard ratio; ICC, intrahepatic cholangiocarcinoma; IRS, immune-related score; LASSO, least absolute shrinkage and selection operator; OS, overall survival; PCa, prostate cancer; prob-KG: probabilistic knowledge graph; pts, patients; RF, random forest; RFE, recursive feature elimination; SVM, support vector machine; UMAP, uniform manifold approximation and projection.

## Data Availability

No new data were created or analyzed in this study. Data sharing is not applicable to this article.
